# Airway stenosis complicated by endobronchial ultrasound‐guided tissue acquisition: A case report

**DOI:** 10.1111/1759-7714.14600

**Published:** 2022-07-27

**Authors:** Keigo Uchimura, Hideaki Furuse, Tatsuya Imabayashi, Yuji Matsumoto, Takaaki Tsuchida

**Affiliations:** ^1^ Department of Endoscopy, Respiratory Endoscopy Division National Cancer Center Hospital Tokyo Japan; ^2^ Department of Thoracic Oncology National Cancer Center Hospital Tokyo Japan

**Keywords:** airway stenosis, bronchoscopy, complication, cryotherapy, endobronchial ultrasound‐guided transbronchial needle aspiration

## Abstract

Endobronchial ultrasound (EBUS)‐guided tissue acquisition (TA) performed by transbronchial needle aspiration (TBNA) is the main diagnostic procedure in mediastinal and hilar lymph node (LN) biopsy. EBUS‐guided intranodal forceps biopsy (EBUS‐IFB) and EBUS‐guided cryobiopsy can achieve higher diagnostic yield of lymphomas, uncommon tumors, and benign diseases. However, these techniques require the creation of a tract to insert biopsy devices, which may result in critical complications. Here, we report a rare case of airway stenosis (AS) that occurred after EBUS‐TA for mediastinal LN biopsy. An 80‐year‐old man had multiple pulmonary nodules and an enlarged mediastinal LN. EBUS‐TBNA and EBUS‐IFB were performed for histological diagnosis. Cutaneous adnexal carcinoma (CAC) was diagnosed. The patient underwent chemotherapy. Four months later, he was hospitalized for AS due to a tracheal tumor with dyspnea. Chest computed tomography and bronchoscopy revealed that the tracheal tumor was caused by invasion from the biopsied LN into the tracheal lumen by tract seeding (TS) caused by EBUS‐TA. Cryotherapy was performed. The tracheal tumor was pathologically consistent with CAC and is currently under control with radiotherapy. TS‐associated EBUS‐TA is rare but may increase in frequency with aggressive tissue sampling techniques. Bronchoscopists should perform EBUS‐TA with awareness of the potentially serious complications.

## Graphical abstract

We report a rare case of airway stenosis that occurred after endobronchial ultrasound (EBUS)‐guided tissue acquisition (TA) for mediastinal lymph node biopsy. Tract seeding associated EBUS‐TA is rare but may increase in frequency with aggressive tissue sampling techniques. Bronchoscopists should perform EBUS‐TA with awareness of the potentially serious complications.

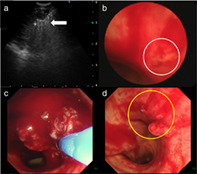

## INTRODUCTION

Endobronchial ultrasound (EBUS)‐guided tissue acquisition (TA) performed by transbronchial needle aspiration (TBNA) is the main diagnostic procedure for mediastinal and hilar lymphadenopathies given its safety and minimal invasiveness.^1^, ^2^, ^3^, ^4^, ^5^, ^6^ While EBUS‐TBNA has high sensitivity and specificity for diagnosing lymph node (LN) metastasis in lung cancer,^1^, ^2^ additional TA with EBUS‐TBNA, such as EBUS‐guided intranodal forceps biopsy (EBUS‐IFB) or EBUS‐guided cryobiopsy (EBUS‐cryo), can achieve higher diagnostic yields of lymphomas, uncommon tumors, and benign diseases, such as sarcoidosis.^7^, ^8^, ^9^, ^10^ EBUS‐IFB and EBUS‐cryo are advantageous as they can help obtain histological tissue samples.^8^, ^9^ However, they require the creation of a tract for inserting forceps or cryoprobes through the tracheal/bronchial wall and LN capsule, which may result in unforeseen complications.

Here, we report a rare case of airway stenosis (AS) that occurred 4 months after EBUS‐TA for mediastinal LN biopsy.

## CASE REPORT

**FIGURE 1:**
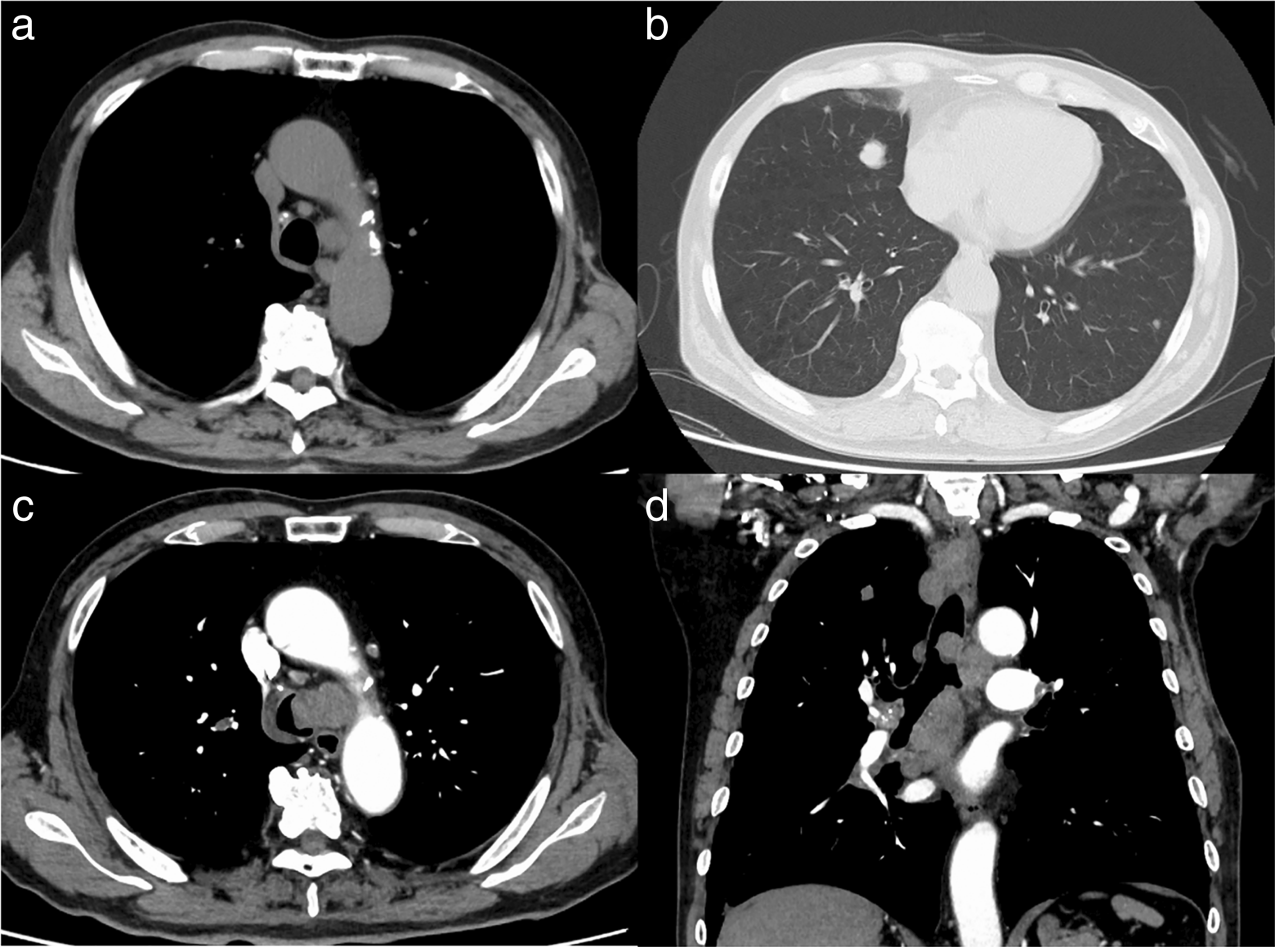
Chest computed tomography (CT) on the initial visit and admission. (a, b) Chest CT on the initial visit showing multiple pulmonary nodules in both lungs and an enlarged lower paratracheal (no. 4 L) lymph node (LN) (a, b; axial image). (c, d) Chest CT on admission showing airway stenosis and a connected tracheal tumor from the bronchoscopically biopsied LN into the tracheal lumen in addition to further mediastinal LN enlargement (c; axial image, d; coronal image)

**FIGURE 2:**
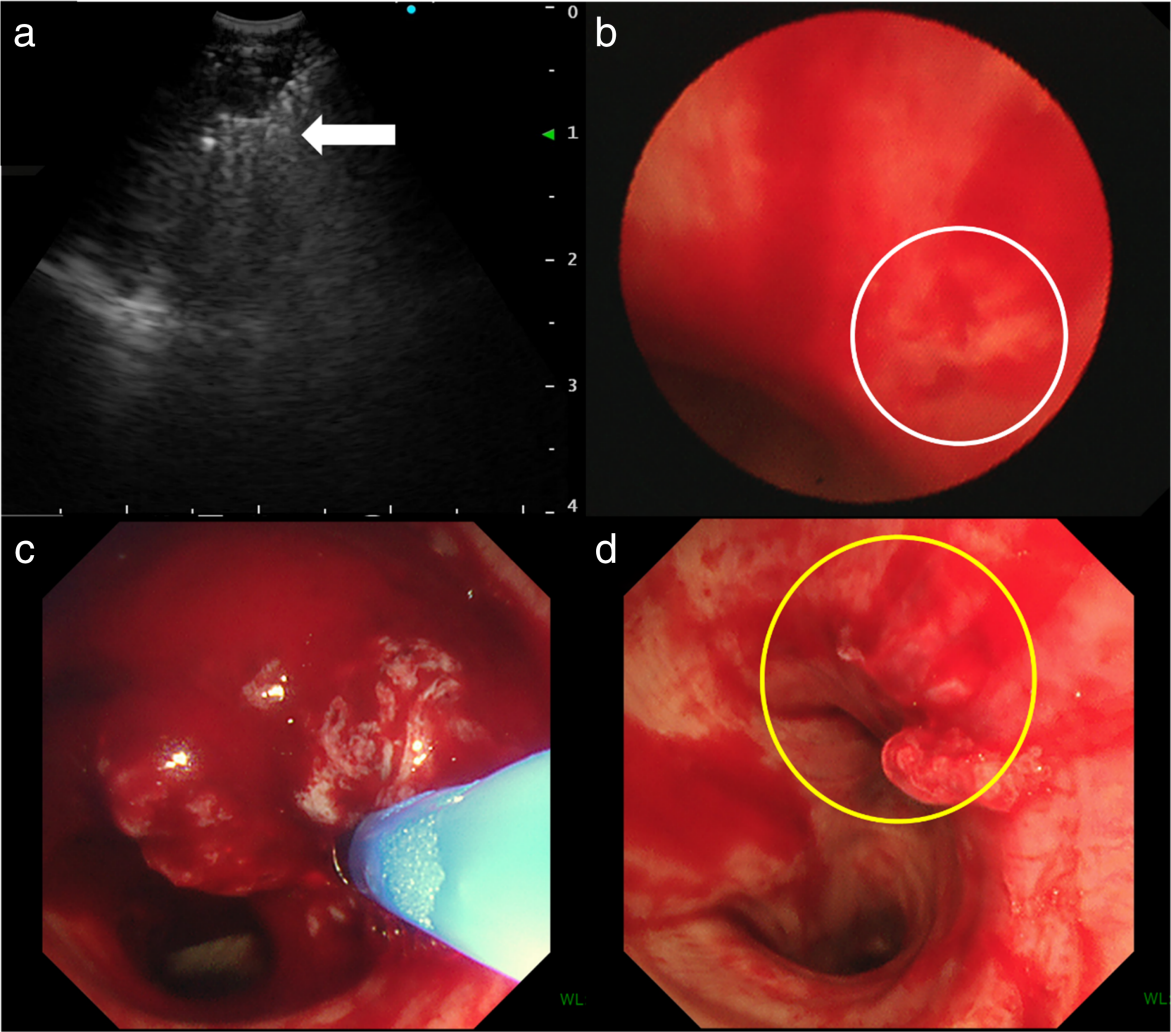
Bronchoscopic findings during diagnostic and therapeutic procedures. (a) An endobronchial ultrasound (EBUS) image during EBUS‐guided intranodal forceps biopsy (EBUS‐IFB) for a mediastinal (no. 4 L) lymph node (white arrow shows opened biopsy forceps within the lymph node). (b) Bronchoscopic findings after EBUS‐IFB (white circle shows the created tract). (c) Bronchoscopic findings of the tracheal tumor. (d) Bronchoscopic findings after securing airway (yellow circle shows the root of the tracheal tumor)

**FIGURE 3:**
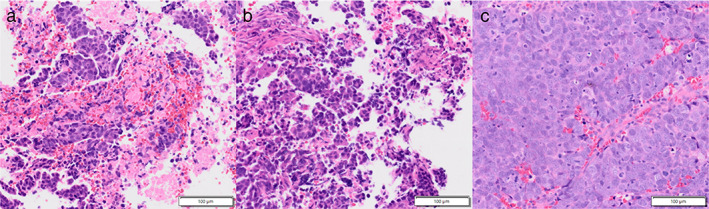
Histopathological findings of the specimens obtained on bronchoscopy. In all specimens (a) transbronchial needle aspiration for the lymph node; (b) forceps biopsy for the lymph node; (c) cryobiopsy for the tracheal tumor, tumor cells with chromatin‐rich, different‐sized nuclei, and eosinophilic cytoplasm are similarly observed, and the specimens were diagnosed as cutaneous adnexal carcinoma (a–c, hematoxylin and eosin staining)

An 80‐year‐old man with a 100 pack‐year smoking history was referred to our hospital for treatment of multiple pulmonary nodules and an enlarged mediastinal LN. He had previously undergone excision of a 3‐cm left axillary tumor, and cutaneous adnexal carcinoma (CAC) was diagnosed. He was medicated for hyperuricemia and reflux esophagitis and had no allergies. His laboratory results were normal, including coagulation test results (Table 1). Chest computed tomography (CT) showed multiple pulmonary nodules in both lungs and an enlarged lower paratracheal LN (Figure 1a,b). LN metastasis from CAC was suspected; hence, EBUS‐TBNA with four punctures made using a convex probe ultrasound bronchoscope (BF‐UC290F, Olympus) and a 22‐gauge needle (EchoTip Ultra, Cook Medical) followed by three EBUS‐IFB procedures using biopsy forceps (FB‐231D, Olympus) was performed on the LN (Figure 2a). Hemostasis from the created tract was confirmed (Figure 2b). The characteristics of the specimen were pathologically consistent with CAC (Figure 3a,b). Chemotherapy was administered. Four months later, the patient was hospitalized with dyspnea. Chest CT showed a connected tracheal tumor from the biopsied LN in addition to further mediastinal LN enlargement (Figure 1c,d). After cryobiopsy and cryotherapy to secure an airway, the root of the tumor was found to be coincident with the tract created by EBUS‐TA (Figure 2b–d). The AS was considered to be caused by tumor invasion into the tracheal lumen by tract seeding (TS) caused by EBUS‐TA. The tracheal tumor was pathologically consistent with CAC (Figure 3c) and is currently under control with radiotherapy.

**TABLE 1:** Patient laboratory data on the initial visit

<Blood cell counts>			<Blood chemistry>			<Tumor marker>		
WBC	4900	/μl	TP	7.4	g/dl	CEA	1.7	ng/ml
Neutrophils	66.8	%	T‐bil	1.1	mg/dl	CA19‐9	11	U/ml
Lymphocytes	24.5	%	AST	25	IU/l	NSE	16	ng/ml
Eosinophils	0.8	%	ALT	18	IU/l	SCC	0.6	ng/ml
Monocytes	7.3	%	LDH	193	IU/l			
Basophils	0.6	%	ALP	57	IU/l	<Coagulation>		
RBC	4.50 × 10^6^	/μl	γ‐GTP	16	IU/l	PT	11	Second
Hb	14.3	g/dl	BUN	4.3	mg/dl	PT%	110	%
Ht	42.3	%	Cre	0.81	mg/dl	PT‐INR	0.95	
Platelets	25.7 × 10^4^	/μl	CRP	0.09	mg/dl	APTT	28	Second

Abbreviations: ALP, alkaline phosphatase; ALT, alanine aminotransferase; APTT, activated partial thromboplastin time; AST, aspartate aminotransferase; BUN, blood urea nitrogen; CA19‐9, carbohydrate antigen 19–9; CEA, carcinoembryonic antigen; Cre, creatinine; CRP, c‐reactive protein; Hb, hemoglobin; Ht, hematocrit; INR, international normalized ratio; LDH, lactate dehydrogenase; NSE, neuron‐specific enolase; PT, prothrombin time; RBC, red blood cell; SCC, squamous cell carcinoma antigen; T‐bil, total bilirubin; TP, total protein; WBC, white blood cell; γ‐GTP, gamma‐glutamyl transferase.

## DISCUSSION

We present a rare AS case caused by TS complicated by EBUS‐TA. The largest retrospective study on EBUS‐TBNA reported a 1.2% incidence of complications, mostly including bleeding and infections without TS.^6^ A recent meta‐analysis reported a higher incidence of complications (4.28%; 19/443 patients), mainly pneumomediastinum (1.1%), pneumothorax (1.1%), and bleeding (0.9%), after EBUS‐IFB than after EBUS‐TBNA alone.^8^ Contrastingly, a randomized trial of EBUS‐cryo among 197 patients reported only two cases of pneumothorax (1.0%) and one case of pneumomediastinum (0.5%).^9^ To our knowledge, no AS case caused by TS complicated by EBUS‐TA has been reported. However, three cases of granuloma formation at the puncture site were reported after EBUS‐TBNA for tuberculous mediastinal lymphadenopathy, suggesting mass formation along the needle tract.^11^, ^12^, ^13^

In the gastrointestinal field, endoscopic ultrasound‐guided TA (EUS‐TA) via gastric and duodenal tracts have been performed worldwide for pancreatic and biliary tract tumors and gastrointestinal submucosal tumors, even before EBUS.^14^ TS associated with EUS‐TA for pancreatic cancer has been reported^15^, ^16^ with a frequency of 0.33% (40/12 109 patients) in a Japanese national survey on resected pancreatic tumor after EUS‐TA.^17^ Three reasons may explain why TS has not been reported in EBUS‐TA but not EUS‐TA. First, unlike the gastrointestinal tract, the tracheal and bronchial walls are firmly supported by cartilages, making it anatomically difficult to invade the airway. Second, most target patients in whom TS may occur have advanced‐stage lung cancer and are not candidates for surgery immediately after diagnosis, proving TS is pathologically difficult using extracted specimens after EBUS‐TA. Third, chemotherapy advances have enabled disease control in most malignancies before causing TS after EBUS‐TA.

However, newer techniques, such as EBUS‐IFB and EBUS‐cryo as well as newer EBUS‐TBNA needles with fabricated needle tips for core tissue sampling and 19‐gauge EBUS‐TBNA needles, may create a firmer and larger tract than the standard 21‐gauge or 22‐gauge EBUS‐TBNA needle.^18^, ^19^, ^20^ There are currently no TS reports with newer techniques or needles for EBUS‐TA. Although newer techniques and needles may be advantageous in tissue sampling, bronchoscopists should be aware of the possibility of tumor invasion through the tract created with EBUS‐TA.

Here, clarifying the main reason for AS after EBUS‐TA was difficult; the involvement of the size of the tract created by EBUS‐TBNA and EBUS‐IFB cannot be ruled out. However, AS due to TS has not been reported, even in EBUS‐cryo,^9^, ^10^ which can help pass larger specimens through the tract than EBUS‐IFB. Therefore, we speculate that characteristics of CAC, which was poorly responsive to chemotherapy, were probably involved. CAC is usually diagnosed on skin biopsy. There is no established chemotherapy; surgical excision and irradiation are the mainstays of treatment. We believe that AS should be considered when performing EBUS‐TA for both CAC and metastatic LNs of tumors with poor response to therapy.

In conclusion, we report an EBUS‐TA‐complicated AS case. Bronchoscopists should perform EBUS‐TA understanding that aggressive tissue sampling can have potentially serious complications.

## CONFLICT OF INTEREST

The authors declare that they have no competing interests.
